# Measures Taken to Prevent Zika Virus Infection During Pregnancy — Puerto Rico, 2016

**DOI:** 10.15585/mmwr.mm6622a2

**Published:** 2017-06-09

**Authors:** Denise V. D’Angelo, Beatriz Salvesen von Essen, Mark J. Lamias, Holly Shulman, Wanda I. Hernandez-Virella, Aspy J. Taraporewalla, Manuel I. Vargas, Leslie Harrison, Sascha R. Ellington, Leslianne Soto, Tanya Williams, Aurea Rodriguez, Carrie K. Shapiro-Mendoza, Brenda Rivera, Shanna Cox, Karen Pazol, Marion E. Rice, Deborah L. Dee, Lisa Romero, Eva Lathrop, Wanda Barfield, Ruben A. Smith, Denise J. Jamieson, Margaret A. Honein, Carmen Deseda, Lee Warner

**Affiliations:** ^1^Division of Reproductive Health, National Center for Chronic Disease Prevention and Health Promotion, CDC; ^2^Division of Maternal, Child, and Adolescent Health, Puerto Rico Department of Health; ^3^Office of Epidemiology and Research, Puerto Rico Department of Health; ^4^Division of Congenital and Developmental Disorders, National Center on Birth Defects and Developmental Disabilities, CDC.

Zika virus infection during pregnancy remains a serious health threat in Puerto Rico. Infection during pregnancy can cause microcephaly, brain abnormalities, and other severe birth defects (*1*). From January 1, 2016 through March 29, 2017, Puerto Rico reported approximately 3,300 pregnant women with laboratory evidence of possible Zika virus infection (*2*). There is currently no vaccine or intervention to prevent the adverse effects of Zika virus infection during pregnancy; therefore, prevention has been the focus of public health activities, especially for pregnant women (*3*). CDC and the Puerto Rico Department of Health analyzed data from the Pregnancy Risk Assessment Monitoring System Zika Postpartum Emergency Response (PRAMS-ZPER) survey conducted from August through December 2016 among Puerto Rico residents with a live birth. Most women (98.1%) reported using at least one measure to avoid mosquitos in their home environment. However, only 45.8% of women reported wearing mosquito repellent daily, and 11.5% reported wearing pants and shirts with long sleeves daily. Approximately one third (38.5%) reported abstaining from sex or using condoms consistently throughout pregnancy. Overall, 76.9% of women reported having been tested for Zika virus by their health care provider during the first or second trimester of pregnancy. These results can be used to assess and refine Zika virus infection prevention messaging and interventions for pregnant women and to reinforce measures to promote prenatal testing for Zika.

The Puerto Rico Department of Health (PRDH), in collaboration with CDC, collected data using a methodology adapted from the Pregnancy Risk Assessment Monitoring System (PRAMS) (*4*) to obtain island-wide and regionally representative information regarding experiences related to prevention and detection of Zika virus infection during pregnancy among women who had a live birth from August 28, 2016 to December 3, 2016. Thirty-six hospitals in Puerto Rico reporting ≥100 births in 2015 (representing >98% of live births) were eligible, and all agreed to participate. Hospitals were assigned to one of eight regional strata corresponding to health districts (Arecibo, Aguadilla, Bayamon, Caguas, Fajardo, Mayaguez, Metro, and Ponce). Regions with fewer births were oversampled to ensure sufficient sample size for computing region-specific estimates. Mothers were selected for inclusion, with probability of selection proportional to the size of the total birth cohort within each region. Within each hospital, clusters (delivery dates) were selected using random sampling. Hospital birth logs were used to identify women (Puerto Rico residents) who gave birth on the selected dates to include in the sample. Sampled women were approached during their hospital stay, 24–36 hours after delivery, and invited to complete a self-administered survey using either a tablet computer (Dell Venue Pro 7139) or paper form. A small incentive (crib mosquito net, calendar of baby’s first year, or mosquito repellent) was offered to participants. Women not contacted before hospital discharge were not followed up. Data were weighted to account for the stratified sampling design and to adjust for differential nonresponse. Percentages and 95% confidence intervals (CIs) were calculated for all indicators. 

Among 2,933 selected women, 2,364 (80.6%) agreed to participate. Among respondents, 72.0% completed the survey via tablet, and 28.0% used the paper form. Most women (79.7%) were aged 20–34 years, 59.5% were married, 68.6% had more than a high school education, 68.6% had Medicaid coverage for prenatal care, 88.5% were recipients of the Special Supplemental Nutrition Program for Women, Infants, and Children, and 91.3% received prenatal care during the first trimester of pregnancy.

Most women reported feeling somewhat or very worried about contracting Zika during their pregnancy (93.4%), and about the possibility of microcephaly or other birth defects in their infants (92.3%) (Table 1). Most women (94.3%) also reported that their health care provider talked to them about Zika virus infection during pregnancy, including counseling them about the risk for transmitting Zika to their baby (91.1%), how to prevent mosquito bites (89.4%), and the use of condoms to prevent sexual transmission of Zika during pregnancy (86.8%). Altogether, 70.6% of women considered their health care provider to be the best source of information about Zika virus infection. Approximately three quarters of respondents reported that their health care provider offered a test (78.2%), and most reported that they were subsequently tested for Zika virus infection (76.9%) during the first or second trimester of pregnancy (Table 1).

**TABLE 1:** Concerns about Zika virus, health care provider counseling and testing, and use of measures to prevent Zika virus transmission during pregnancy among Puerto Rico residents with a recent live birth — Pregnancy Risk Assessment Monitoring System Zika Postpartum Emergency Response Survey, Puerto Rico, 2016

Survey responses	Overall total (n = 2,364)	
	Unweighted no.*	%^†^ (95% CI)
**Maternal Zika-related concern**		
Somewhat/Very worried about getting Zika	2,205	93.4 (92.4–94.3)
Somewhat/Very worried about microcephaly/birth defects in baby	2,154	92.3 (91.2–93.2)
**Information from health care provider**		
Counseling on Zika virus (any discussion)	2,155	94.3 (93.4–95.0)
Counseling on risk for passing Zika virus to baby	2,053	91.1 (90.0–92.0)
Counseling on how to prevent mosquito bites	2,030	89.4 (88.2–90.5)
Counseling on using condoms to prevent sexual transmission of Zika	1,982	86.8 (85.4–88.1)
Considered health care provider best source of information on Zika	1,662	70.6 (68.7–72.3)
**Testing for Zika**		
Health care provider offered Zika test in first or second trimester of pregnancy	1,801	78.2 (76.6–79.7)
Health care provider provided Zika test in first or second trimester of pregnancy	1,758	76.9 (75.3–78.4)
**Use of measures to prevent Zika virus infection**		
**Environmental measures to avoid mosquito bites**		
Always used screens on open doors and open windows, or always kept unscreened doors and windows closed	2,032	88.4 (87.0–89.6)
Removed standing water from around the home weekly	2,068	88.7 (87.3–90.0)
Received professional indoor/outdoor spraying of home	1,274	55.0 (53.1–56.9)
Received professional larvicide application outside home	688	29.3 (27.5–31.1)
Slept under mosquito net	424	17.4 (16.0–18.9)
**Personal measures to avoid mosquito bites**		
Wore long sleeves and pants every day	262	11.5 (10.3–12.8)
Used mosquito repellent every day	1,055	45.8 (43.9–47.8)
**Measures to prevent sexual transmission**		
Abstained from sexual activity for entire pregnancy for any reason	467	19.9 (18.4–21.5)
Condom use during pregnancy among sexually active women (n = 1,864)		
Every time	414	22.7 (20.8–24.6)
Sometimes^§^	372	21.2 (19.4–23.1)
Never	1,017	56.2 (53.9–58.4)
**Measures to prevent mosquito bites and sexual transmission**		
Used at least one environmental protective measure around the home	2,309	98.1 (97.4–98.6)
Used at least one personal protective measure every day (long sleeves and pants or repellent)	1,128	48.8 (46.9–50.8)
Used at least one measure to avoid sexual transmission for entire pregnancy (sexual abstinence or condom use)	881	38.5 (36.6–40.4)
Used at least one personal protective measure against mosquitos and at least 1 personal protective measure against sexual transmission consistently throughout pregnancy	552	24.2 (22.6–26.0)

**Abbreviation**: CI = confidence interval.

* Unweighted sample size; sample size varies because of missing responses or skip pattern in survey.

^†^ Weighted percent.

^§^ Excludes condom use every time.

Measures to prevent mosquito bites in the home environment were reported to be commonly practiced during pregnancy. These included always using screens on windows and doors or keeping unscreened windows and doors closed (88.4%); removing standing water from the house and yard weekly (88.7%); receiving professional indoor/outdoor spraying of the home (55.0%); and receiving professional larvicide application outside the home (29.3%). Fewer than two in 10 women (17.4%) reported sleeping under a mosquito net at some time during pregnancy. Overall, 98.1% of women adopted at least one measure to protect their home environment from mosquitos (Table 1).

Use of personal protective measures against mosquito bites was reported less frequently than implementation of home environment prevention measures. Personal protective measures included wearing long-sleeved shirts and pants daily during pregnancy (11.5% of participants), and using mosquito repellent on exposed skin every day when outside (45.8%) (Table 1). Being too hot (76.4%) was the most commonly reported reason for not wearing long-sleeved shirts and pants. Forgetting to apply/reapply repellent (51.4%), disliking the smell (18.8%), and being concerned that chemicals would harm the baby (15.3%) were the most commonly reported reasons for not using repellent daily (Table 2).

**TABLE 2:** Reasons for not using measures to prevent Zika virus transmission during pregnancy among Puerto Rico residents with a recent live birth — Pregnancy Risk Assessment and Monitoring System Zika Postpartum Emergency Response Survey, Puerto Rico, 2016

Survey response	Unweighted no.*	%^†^ (95% CI)
**Reason for not wearing long sleeves and pants every day (n = 2339)**		
It was too hot to wear long sleeves or long pants	1,783	76.4 (74.7–78.0)
My clothes with long sleeves or long pants no longer fit because of pregnancy	457	19.7 (18.1–21.3)
I did not have clothes with long sleeves or long pants	111	4.7 (4.0–5.7)
I was indoors	44	2.1 (1.6–2.8)
Some other reason	131	5.3 (4.5–6.2)
**Reasons for not using mosquito repellent every day on exposed skin when outside (n = 2241)**		
I forgot to apply/reapply it	1,137	51.4 (49.4–53.4)
I did not like the way it smelled	408	18.8 (17.3–20.4)
I worried about the chemicals in the repellent harming my baby	329	15.3 (13.9–16.9)
I did not like the way it made my skin feel	265	11.8 (10.6–13.1)
I worried about the chemicals in the repellent harming me	128	5.8 (4.9–6.8)
I was indoors	56	2.7 (2.1–3.5)
Mosquito repellent was too expensive	54	2.5 (1.9–3.2)
I have an allergy	35	1.4 (1.0–1.8)
Some other reason	200	7.9 (6.9–9.0)
**Reasons for not using condoms every time during sex**^§^ **(n = 1406)**		
I didn't think my partner had Zika virus	505	37.4 (35.0–39.9)
I didn't think I needed to use condoms during pregnancy	432	31.8 (29.5–34.3)
I didn't want to use condoms	287	20.1 (18.2–22.2)
I forgot to use condoms	180	12.2 (10.6–13.9)
My partner didn't want to use condoms	153	11.0 (9.5–12.7)
I didn't know you could get Zika virus from having sex	94	7.3 (6.0–8.9)
I didn't think a condom would prevent Zika infection	58	4.7 (3.7–6.0)
I was not worried about getting the Zika virus	41	3.1 (2.3–4.2)
I could not get condoms when I needed them	33	2.3 (1.7–3.2)
Allergy	29	2.1 (1.5–3.0)
I could not afford condoms	20	1.6 (1.0–2.4)
Some other reason	105	7.1 (6.0–8.5)

**Abbreviation**: CI = confidence interval.

* Unweighted sample size; sample size varies because of missing response or skip pattern in survey.

^†^ Weighted percent.

^§^ Among women who were sexually active during pregnancy.

Measures to prevent sexual transmission of Zika virus during pregnancy through sexual abstinence or consistent condom use were not commonly practiced; overall, one in five (19.9%) women reported that they abstained from sex during the entire pregnancy, one quarter of whom did so specifically to avoid Zika virus infection. Among sexually active women, less than a quarter (22.7%) reported using condoms consistently throughout pregnancy. Altogether, approximately one third (38.5%) of respondents reported using at least one measure to prevent sexual transmission of Zika during pregnancy (abstinence or consistent condom use) (Table 1). Common reasons for not using condoms consistently were not thinking her partner had Zika (37.4%), not thinking that condom use was necessary during pregnancy (31.8%), and not wanting to use condoms (20.1%) (Table 2).

Overall, approximately one quarter (24.2%) of women reported using at least one personal protective measure against mosquito bites daily (repellent or protective clothing) and at least one protective measure against sexual transmission (abstinence or consistent condom use) throughout pregnancy.

## Discussion

In 2016 and early 2017, approximately 3,300 pregnant women in Puerto Rico had laboratory evidence of possible Zika virus infection, the largest number in the United States (*2*,*5*). Because Zika virus infection during pregnancy can cause microcephaly and other severe brain defects in infants (*1*,*6*), public health measures have focused on raising awareness about the virus and ways to prevent infection among pregnant women.*^,^^†^^,^^§^ PRDH has widely disseminated information to the public regarding avoiding mosquito bites to prevent Zika virus infection. The messaging might be familiar to many women because similar guidance has been provided in earlier campaigns to prevent mosquito-borne illnesses such as dengue and chikungunya (*7*). Whereas almost all respondents to this survey (98.1%) used environmental measures to protect themselves from mosquito bites around the home, reported use of personal protective measures was less common (<50%), despite respondent awareness of and concern about the risks of contracting Zika virus infection during pregnancy. Public awareness campaigns and provider advice should focus on prevention messages related to the safety of repellent use during pregnancy, and include specific suggestions about remembering repellent use daily and the availability of scent-free repellent.

The CDC Foundation, with technical assistance from CDC and in partnership with PRDH, has launched initiatives promoting the prevention of transmission of Zika virus infection during pregnancy, including sexual transmission, through engagement of social networks and communities surrounding pregnant women (*8*).^¶^ The findings from this study, however, suggest that measures to improve adherence to recommendations about prevention of sexual transmission need reinforcement. Although respondents reported high levels of concern about Zika virus infection and of receipt of counseling by health care providers about using condoms to prevent sexual transmission, only approximately one in five women reported consistently using condoms throughout pregnancy. The most common reasons for not using condoms were thinking that their partner did not have Zika virus and thinking that condoms were not needed during pregnancy. Given that CDC guidance recommends that pregnant women in areas with risk for Zika virus infection abstain from sexual intercourse or consistently use condoms during pregnancy,** the results from this study point to the need to further evaluate the content of health care provider counseling and communication campaigns, and to identify personal barriers to condom use to ensure that the ongoing risk for Zika virus infection through sexual transmission during pregnancy is clearly understood by women and their male partners.^††^

In February 2016, CDC issued recommendations that all symptomatic and asymptomatic pregnant women in regions with ongoing Zika virus transmission be tested for infection at the initiation of prenatal care, with follow-up testing in the second trimester (*9*). These recommendations were adopted immediately by the PRDH with the release of administrative orders prompting health care providers to offer testing to pregnant women. Nevertheless, >20% of survey respondents reported not receiving testing for Zika virus infection in their first or second trimester, indicating a need for enhanced measures to increase awareness and implementation of the testing guidelines for pregnant women. PRDH modified the testing guidance on October 19, 2016, and added a requirement for third trimester testing (*10*). Although prenatal testing could be improved, findings from this survey demonstrate that health care providers are adhering to recommendations to counsel pregnant women about Zika virus infection (>90% of respondents reported receipt of counseling).

The findings in this report are subject to at least three limitations. First, data do not represent all pregnant women in Puerto Rico in 2016; only live births from late August through early December were included. Given that the height of the Zika outbreak in Puerto Rico was in August, the group of surveyed women might differ in their behaviors from women who became pregnant later in the outbreak. Second, women whose pregnancy did not result in a live birth, who gave birth in hospitals with fewer than 100 births annually, or who gave birth outside the hospital setting were not included. Finally, this self-reported information is subject to social desirability bias on sensitive topics such as sexual activity and condom use, and recall bias for preventive behaviors practiced throughout pregnancy, which could have resulted in misreporting of these behaviors.

Understanding health behaviors of pregnant women during the Zika outbreak can inform programs and initiatives that seek to prevent Zika virus infection and promote testing of pregnant women in Puerto Rico. In particular, these data illuminate gaps in the use of preventive measures that could be reinforced during prenatal care visits and through public communication campaigns. Messages pertaining to the safety and frequency of use of mosquito repellent, the need for sexual abstinence or consistent condom use during pregnancy, and provider adherence to recommended testing guidelines for Zika virus infection can improve the prevention and detection of Zika virus infection during pregnancy.

**Summary**

**What is known about this topic?**

Zika virus infection during pregnancy can cause microcephaly, brain abnormalities, and other severe birth defects. Puerto Rico has recorded the largest number of laboratory-confirmed cases of Zika virus infections among pregnant women in the United States, and has implemented strategies to prevent infection during pregnancy and ensure health care provider counseling and testing for Zika virus.

**What is added by this report?**

Among women in Puerto Rico who had a recent live birth, 98.1% reported using at least one measure to avoid mosquitos in their home environment during their pregnancy. However, fewer than half of women reported wearing mosquito repellent daily (45.8%), and only one in 10 reported wearing pants and shirts with long sleeves daily. Among sexually active pregnant women, 38.5% reported abstaining from sex or using condoms consistently throughout pregnancy. Most women (94.3%) also reported that their health care provider talked to them about Zika virus infection during pregnancy, and approximately three quarters of respondents (76.9%) reported being tested for Zika virus by their health care provider during the first or second trimester of pregnancy.

**What are the implications for public health practice?**

Women in Puerto Rico have high levels of concern about acquiring Zika virus infection during pregnancy, and health care providers are counseling them about Zika virus prevention. However, additional measures are needed to encourage consistent use of preventive measures throughout pregnancy and increase testing for Zika virus during pregnancy.

## Footnotes

* https://www.cdc.gov/zika/prevention/index.html.

† http://www.salud.gov.pr/Sobre-tu-Salud/Pages/Condiciones/Zika.aspx.

§ https://www.hhs.gov/sites/default/files/hhs-assistance-to-puerto-rico-to-fight-zika.pdf.

¶ http://detenelzika.org/.

** https://www.cdc.gov/mmwr/volumes/65/wr/mm6539e1.htm?s_cid=mm6539e1_w.

†† https://www.cdc.gov/condomeffectiveness/.
